# The Efficacy of *Panax ginseng* for the Treatment of Nonalcoholic Fatty Liver Disease: A Systematic Review and Meta-Analysis of Preclinical Studies

**DOI:** 10.3390/nu15030721

**Published:** 2023-01-31

**Authors:** Keungmo Yang, Hee-Hoon Kim, Young-Ri Shim, Myeong Jun Song

**Affiliations:** 1 Department of Internal Medicine, Division of Gastroenterology and Hepatology, College of Medicine, The Catholic University of Korea, Seoul 06591, Republic of Korea; 2 Life Science Research Institute, Korea Advanced Institute of Science and Technology, Daejeon 34141, Republic of Korea

**Keywords:** meta-analysis, *Panax ginseng*, nonalcoholic fatty liver disease, liver injury, dyslipidemia, glucose tolerance

## Abstract

Although tremendous research has reported the protective effects of natural compounds in nonalcoholic fatty liver disease (NAFLD), there is still no approved drug. This study aimed to examine the efficacy of *Panax ginseng* in NAFLD in preclinical studies. A total of 41 studies were identified by searching the PubMed, Web of Science, and Cochrane Library databases. The methodological quality was assessed by the risk of bias tool from the Systematic Review Center for Laboratory Animal Experimentation. The standardized mean difference (SMD) with a 95% confidence interval was calculated, and the random effects model was used to examine overall efficacy or heterogeneity. The publication bias was analyzed by Egger’s test. The results showed that *Panax ginseng* treatment significantly reduced the systemic levels of alanine aminotransferase (SMD: −2.15 IU/L; *p* < 0.0001), aspartate aminotransferase (SMD: −2.86 IU/L; *p* < 0.0001), triglyceride (SMD: −2.86 mg/dL; *p* < 0.0001), total cholesterol (SMD: −1.69 mg/dL; *p* < 0.0001), low-density lipoprotein (SMD: −1.46 mg/dL; *p* < 0.0001), and fasting glucose (SMD: −1.45 mg/dL; *p* < 0.0001) while increasing high-density lipoprotein (SMD: 1.22 mg/dL; *p* = 0.0002) in NAFLD regardless of animal models or species. These findings may suggest that *Panax ginseng* is a promising therapeutic agent for NAFLD treatment.

## 1. Introduction

Nonalcoholic fatty liver disease (NAFLD), the most prevalent chronic liver disease worldwide, develops into a continuous spectrum of hepatic dysfunction ranging from nonalcoholic fatty liver (NAFL) to nonalcoholic steatohepatitis (NASH), leading to fibrosis and hepatocellular carcinoma (HCC) [1]. In 1988, Day and James proposed a “two-hit” theory to describe the pathogenesis of NASH [2]. In this theory, intrahepatic triglyceride accumulation is regarded as a “first hit” that makes the liver more vulnerable to injury induced by “second hits”, including inflammation, oxidative stress, and mitochondrial dysfunction. Unlike this “two-hit” theory, recent studies have emphasized the multifaceted features of NAFLD pathogenesis, providing a more accurate description. This “multiple hit” hypothesis suggests that multiple insults, such as insulin resistance, adipokines, and genetic factors, act together to induce NAFLD [3]. In line with this, the development of NAFLD is strongly associated with metabolic syndrome, including obesity, insulin resistance, type 2 diabetes mellitus, and dyslipidemia [4]. Although the recent obesity epidemic and increased sedentary behaviors caused by the COVID-19 pandemic have accelerated the incidence rate of NAFLD worldwide [4,5], there is still no approved drug for the disease, and the first line of therapy is a lifestyle intervention [6]. Therefore, there is an urgent need for a new drug that can cover the various pathogenic mechanisms of NAFLD.

*Panax ginseng* (C. A. Meyer) is an ancient and famous botanical medicine in eastern countries [7]. Currently, approximately 300 substances have been identified in *Panax ginseng*, including ginsenosides, polysaccharides, polyacetylenes, amino acids, and peptides [8]. The previous reports have demonstrated the protective roles of *Panax ginseng* in animal models of liver diseases, cardiovascular diseases, diabetes mellitus, and neurological diseases with its anti-oxidative, anti-inflammatory, and anti-microbial effects [9,10]. In addition, the oral administration of *Panax ginseng* to healthy individuals or Sprague Dawley rats is well-tolerated for 24 weeks or 12 months, respectively [11,12]. Based on the above findings and the support of systematic reviews and meta-analyses, more than 100 clinical trials with *Panax ginseng* that target metabolic syndrome, neurological diseases, and cardiovascular diseases have been registered in the World Health Organization international clinical trials registry platform [13,14,15,16]. However, to date, there is still a lack of clinical trials to study the effects of *Panax ginseng* in patients with NAFLD. In this aspect, a rigorous review based on animal studies may offer solid evidence for future clinical trials.

Here, the present study reports a systematic review and meta-analysis of data obtained from animal models of NAFLD that examined the efficacy of various doses and treatment durations of *Panax ginseng*. In particular, this study focused on the changes in alanine aminotransferase (ALT), aspartate aminotransferase (AST), triglyceride (TG), total cholesterol (TC), high-density lipoprotein (HDL) cholesterol, low-density lipoprotein (LDL) cholesterol, and fasting blood glucose levels by *Panax ginseng* treatment. In addition, the current study explored, through subgroup analysis, whether these outcomes differed to the experimental models or animal species used.

## 2. Materials and Methods

### 2.1. Search Strategy

The present meta-analysis was conducted according to the Preferred Reporting Items for Systematic Reviews and Meta-Analyses (PRISMA) guidelines by two independent researchers [17]. The study protocol was registered in the International Prospective Register of Systematic Reviews (PROSPERO) (registration number: CRD42023381574). From inception to February 2023, PubMed, Web of Science, and the Cochrane Library databases were utilized to search the reliable literature on the efficacy of *Panax ginseng* for the treatment of NAFLD. “Ginseng” and “Fatty liver” were used as the main keywords. The detailed search strategy for the literature was described in Table 1.

### 2.2. Inclusion and Exclusion Criteria

The inclusion criteria for the present study were as follows: (1) The participants were animal models of NAFLD; (2) NAFLD-induced animals were divided into the experiment (with *Panax ginseng* treatment) and control (without *Panax ginseng* treatment) groups; (3) studies included at least one serological marker (ALT, AST, TG, TC, HDL, LDL, or fasting blood glucose) reflecting the severity of NAFLD; (4) studies were published in English; (5) all animal experiments should be approved by the Institutional Animal Care and Use Committee.

Studies containing the following criteria were excluded from the final analysis: (1) Duplicated literature identified from the different searching databases; (2) studies that were not related to the therapeutic efficacy of *Panax ginseng* in NAFLD; (3) studies that used natural products other than *Panax ginseng*; (4) studies without animal experiments; (5) review articles or clinical trials; (6) insufficient data for the primary outcomes; (7) in vitro experiments; and (8) conference abstracts, books, or theses.

### 2.3. Data Extraction

The extraction of the elementary data from included studies was conducted by two independent reviewers as follows: (1) the first author’s name and publication year; (2) the authors’ country; (3) sample size in each group; (4) animal species; (5) diet model of NAFLD and feeding period; (6) components of *Panax ginseng*; (7) treatment dose, route, and times. Digitizing software was utilized to extract the data presented in graphs. If there were discrepancies in the data extraction by the two reviewers, they were resolved after the discussion with the third reviewer.

### 2.4. Quality Assessment

The methodological quality of the included studies was assessed according to the risk of bias tool from the Systematic Review Centre for Laboratory Animal Experimentation (SYRCLE) for animal studies [18]. Categories for the investigation of quality were as follows: (1) Sequence generation; (2) baseline characteristics; (3) allocation concealment; (4) random housing; (5) blinding for the performance bias; (6) random outcome assessment; (7) blinding for the detection bias; (8) incomplete outcome data; (9) selective outcome data; and (10) other sources of bias. Assessment of each category was divided into high, low, or unclear risk of bias.

### 2.5. Statistical Analysis

The present meta-analysis was performed with ReviewManager (RevMan 5.4; London, UK) and R software (version 4.2.1; R foundation, Inc. (Indiana, IN, USA); http://cran.r-project.org (accessed on 1 December 2022). The standardized mean difference (SMD) with a 95% confidence interval (CI) was calculated to investigate the efficacy of *Panax ginseng*. The heterogeneity among included studies was evaluated by the *I*^2^ statistic and Cochrane’s Q-square test. A fixed- or random-effects model was conducted for minor (*I*^2^ ≤ 50% or *p* ≥ 0.1) or major heterogeneity (*I*^2^ > 50% or *p* < 0.1), respectively. Publication bias was analyzed by Egger’s linear regression test. A *p* value < 0.05 was regarded as statistically significant.

## 3. Results

### 3.1. Study Identification and Selection

**Figure 1:**
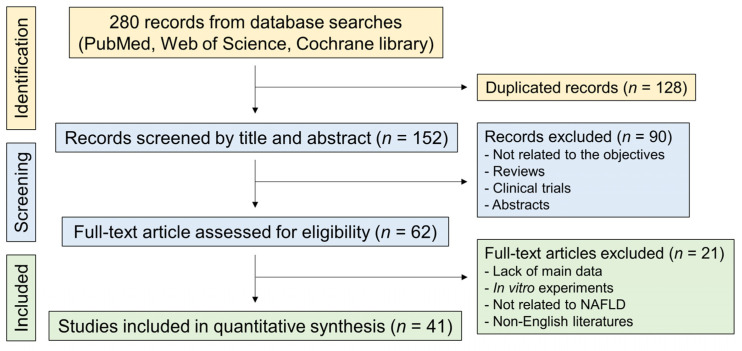
Flow diagram of the systematic literature search. NAFLD, nonalcoholic fatty liver disease.

This study selected reliable literature for meta-analysis according to the flow described in Figure 1. A total of 280 records were initially screened from the PubMed, Web of Science, and Cochrane Library databases, and 128 duplicates were removed. According to the prescribed exclusion criteria, 90 studies that were not related to the present study, review articles, or clinical trials were additionally removed based on the contents of their titles and abstracts. After a careful review of 62 full-text articles, a total of 41 studies were included in the final analysis (Figure 1).

### 3.2. Study Characteristics

All of the included articles (41 studies) were published between 2009 and 2023, and the nationality of the authors was mainly Chinese (53.7%) or Korean (41.5%). The animal species used in the experiments were mice (73.2%) or rats (26.8%). In 24 out of 30 studies that used mice as experimental models, C57BL/6J or C57BL/6N mice were utilized. Other studies used Kunming mice, *db*/*db* mice, or institute of cancer research (ICR) mice. For rat studies, 9 out of 11 studies incorporated Sprague-Dawley rats, and Otsuka Long-Evans Tokushima fatty (OLETF) rats were used in the remaining 2 studies. To induce NAFLD, various animal models, including high-fat diet (HFD), methionine-choline deficient (MCD) diet, high-fat high-sugar (HFHS) diet, or *db*/*db* mice, were used. Treatment agents ranged from a single component, including ginsenosides Rb1, Rg1, or Rh1, to *Panax ginseng* itself. The route of drug administration was mainly oral gavage, and in four studies, drugs were intraperitoneally injected. The administered doses of *Panax ginseng* varied greatly, ranging from 0.4 mg kg^−1^ day^−1^ to 1000 mg kg^−1^ day^−1^. In addition, the treatment period was also diverse, ranging from 3 days to 16 weeks. For the analyses of the outcomes, 30 studies (ALT), 26 studies (AST), 32 studies (TG), 29 studies (TC), 18 studies (HDL), 19 studies (LDL), and 14 studies (fasting blood glucose) were involved. The overall experimental characteristics of the involved studies were summarized in Table 2.

### 3.3. Quality Assessment

**Figure 2:**
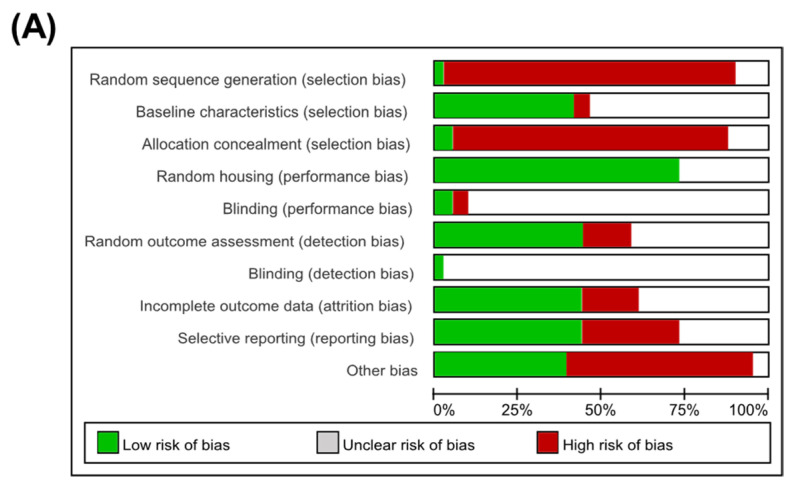

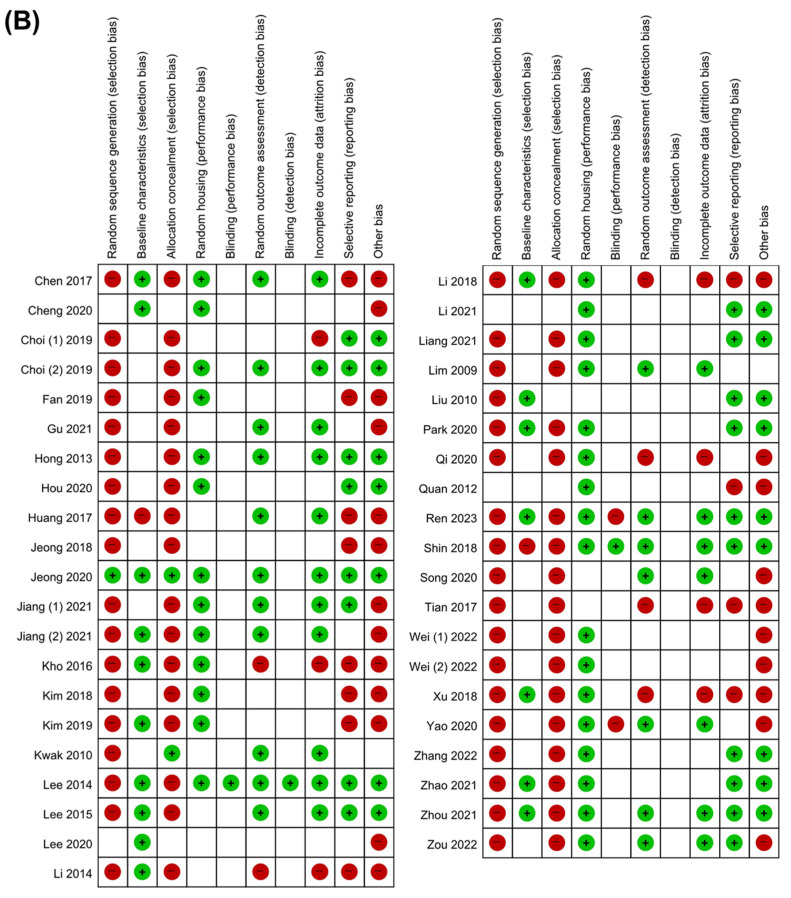
Quality assessment of included studies based on the SYRCLE’s risk of bias tool. (**A**) A graph of the risk in the bias of included studies. (**B**) A summary of the risk of bias in the included studies [7,19,20,21,22,23,24,25,26,27,28,29,30,31,32,33,34,35,36,37,38,39,40,41,42,43,44,45,46,47,48,49,50,51,52,53,54,55,56,57,58].

In quality assessment, only one study conducted random sequence generation. In addition, about 50% (17 of 41) of studies were analyzed with the same baseline characteristics among experimental groups, and two studies had an allocation with concealment. Among the referred studies, random housing of animals was performed in 30 studies. Due to the characteristics of animal experiments, it was difficult to evaluate blinding bias in most studies. The attrition bias was low in 18 studies that had complete outcome data, and they also showed a low risk of reporting bias. Although the overall quality of the referred articles was not satisfactory, no literature was excluded from this process. Detailed information about the quality assessment of the included studies is presented in Figure 2.

### 3.4. Primary Outcomes

#### 3.4.1. Markers for Liver Injury

**Figure 3:**
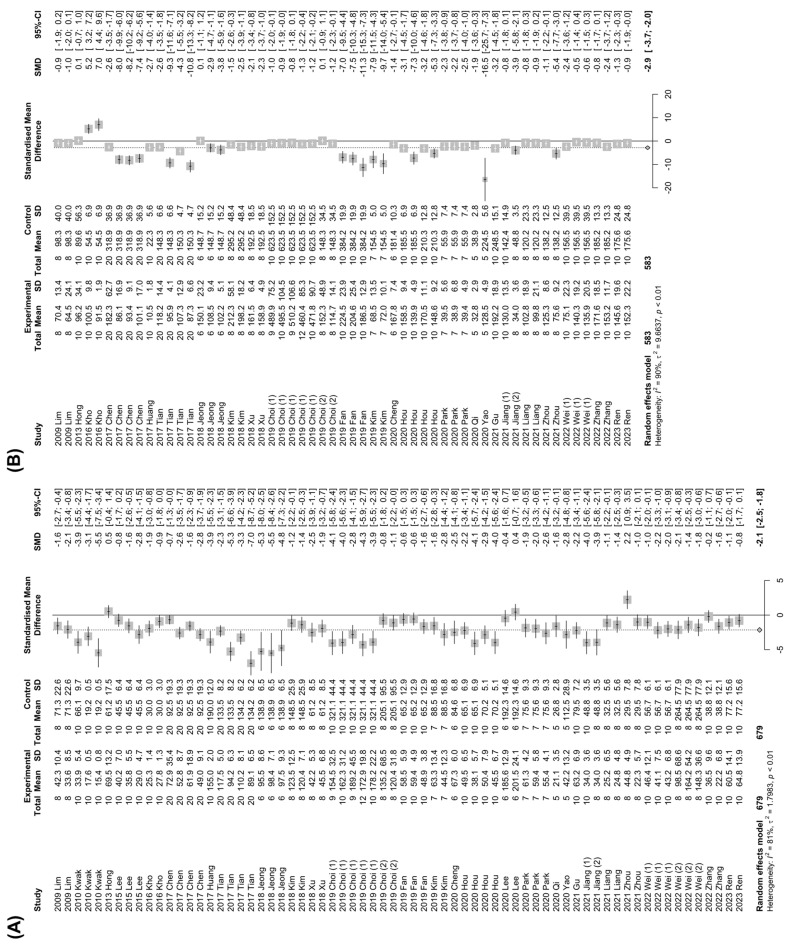
The effect of *Panax ginseng* on the levels of liver injury markers in NAFLD-induced animals. Forest plots for comparison: (**A**) Alanine aminotransferase (ALT) levels [7,19,20,23,24,27,28,29,30,31,32,35,36,37,38,39,40,41,43,44,45,47,48,49,50,52,54,55,56,58]. (**B**) Aspartate aminotransferase (AST) levels [19,23,27,28,29,30,31,32,35,36,37,38,39,40,41,44,45,47,48,49,50,52,54,55,56,58]. SD, standard deviation.

Persistent overload of nutrients and inflammatory cues instigate liver injury, one of the cardinal features of NAFLD [1]. Therefore, the effects of *Panax ginseng* on the systemic levels of liver injury markers (ALT and AST) in animal models of NAFLD were examined. In the analysis of ALT, a total of 30 studies with 1358 animals were analyzed (Figure 3A). Since the results showed a high degree of heterogeneity between the experimental and control groups (*I*^2^ = 81%, *p* < 0.01), the random-effects model was selected for further investigation (Figure 3A). Interestingly, a notable decrease in ALT levels by the *Panax ginseng* treatment was found in NAFLD-induced animals (SMD: −2.15 IU/L; 95% CI: −2.50 to −1.79 mg/dL; *p* < 0.0001) (Figure 3A). Next, to explore the effects of *Panax ginseng* on AST levels, 26 studies with 1166 animals that reported AST levels were analyzed with strong heterogeneity (*I*^2^ = 90%, *p* < 0.01) (Figure 3B). Similar to the results from ALT, *Panax ginseng* administration significantly reduced AST levels in NAFLD-induced animals (SMD: −2.86 IU/L; 95% CI: −3.70 to −2.02 mg/dL; *p* < 0.0001) (Figure 3B). These results may suggest that *Panax ginseng* could attenuate NAFLD-induced liver injury in a preclinical model.

#### 3.4.2. Markers for Hepatic Lipid Metabolism

**Figure 4:**
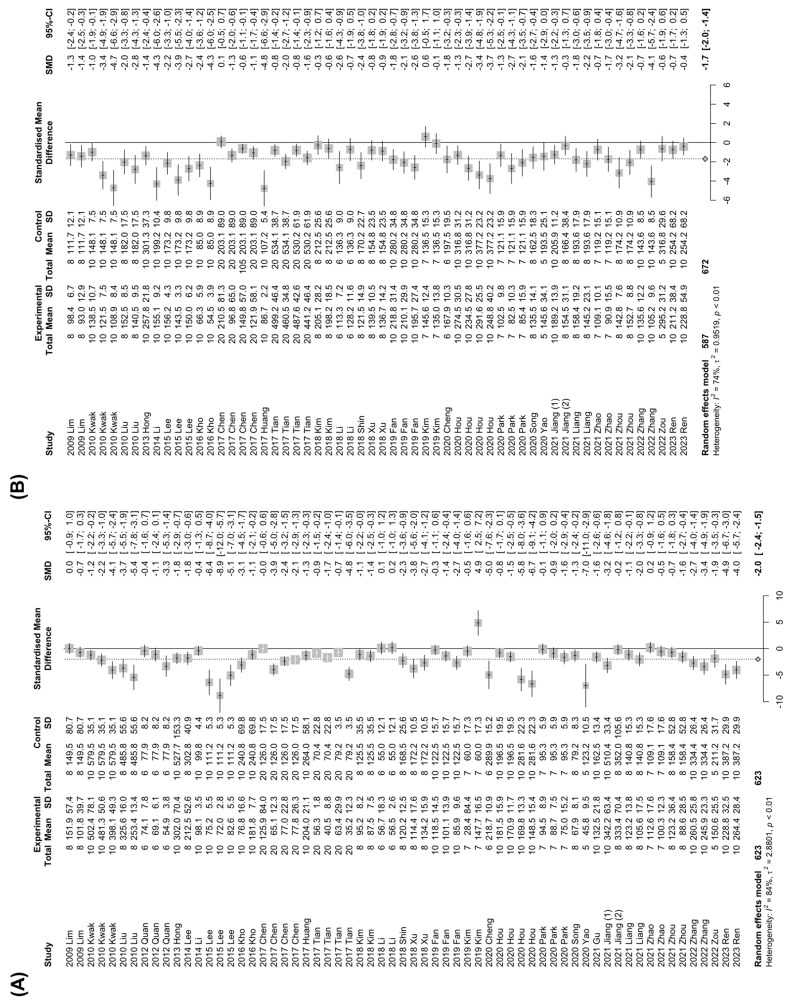

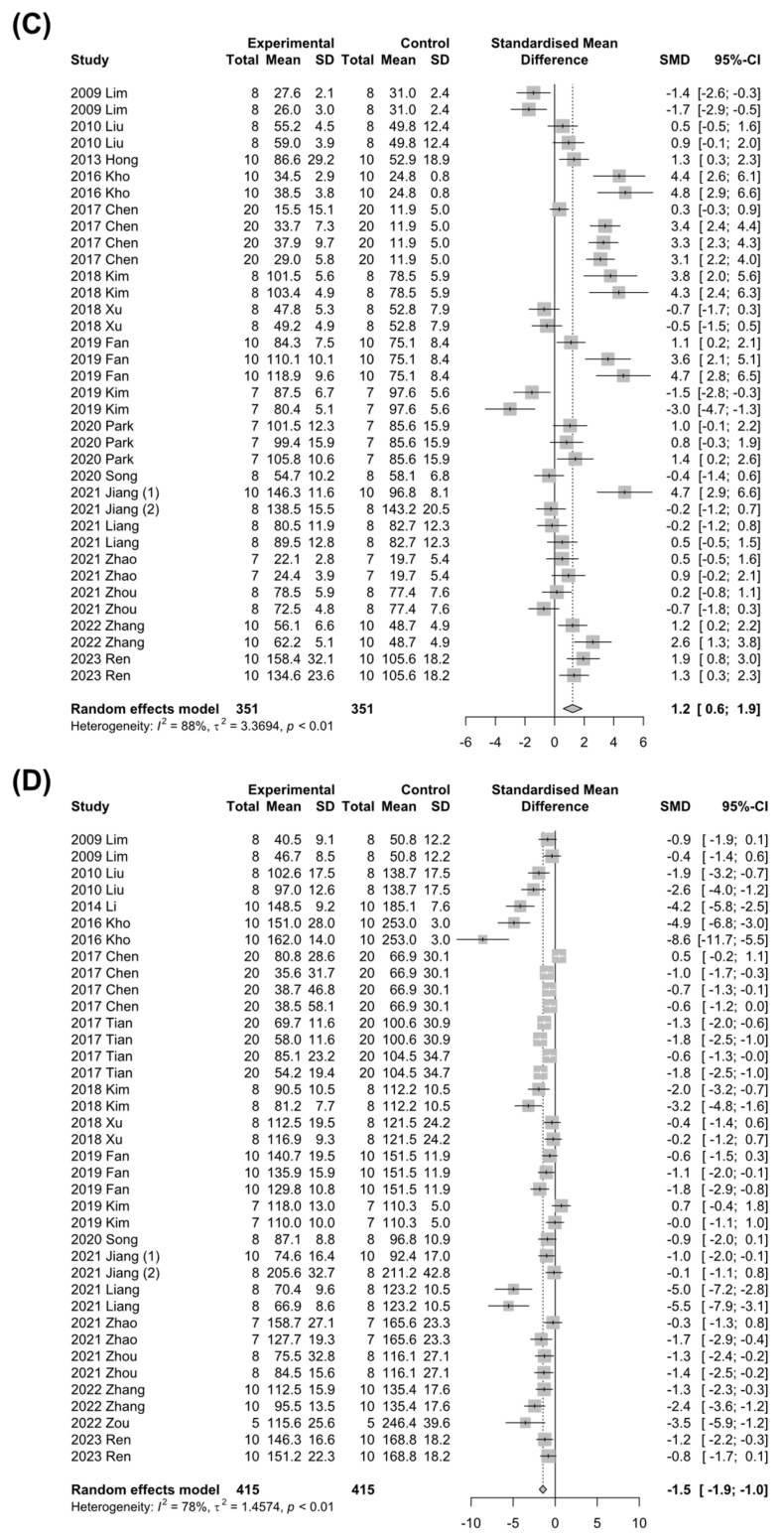
The effect of *Panax ginseng* on hepatic lipid metabolism in NAFLD-induced animals. Forest plots for comparison: (**A**) Triglyceride (TG) levels [7,19,20,21,22,23,24,25,26,27,28,29,30,32,33,34,35,38,39,40,41,44,46,47,48,49,50,51,52,56,58]. (**B**) Total cholesterol (TC) levels [7,19,20,21,23,25,26,27,28,29,30,32,33,34,35,38,39,40,41,44,46,47,49,50,51,52,56,57,58]. (**C**) High-density lipoprotein (HDL) levels [7,19,21,23,27,28,32,35,38,39,44,46,49,50,51,52,56,58]. (**D**) Low-density lipoprotein (LDL) levels [7,19,21,25,27,28,30,32,35,38,39,46,49,50,51,52,56,57,58]. SD, standard deviation.

In the development of NAFLD, dysregulation of hepatic lipid metabolism is a characterized phenotypic change, accompanying an abnormal increase in the systemic TG, TC, and LDL levels, while HDL levels are decreased [1]. Therefore, studies that involved changes in TG, TC, LDL, or HDL levels by *Panax ginseng* treatment in NAFLD-induced animals were investigated. A total of 32 studies with 1246 animals or 29 studies with 1259 animals were analyzed for the efficacy of Panax ginseng on TG or TC levels, respectively. Of note, the *Panax ginseng* treatment significantly lowered TG (SMD: −2.86 mg/dL; 95% CI: −3.70 to −2.02 mg/dL; *p* < 0.0001) and TC (SMD: −1.69 mg/dL; 95% CI: −1.99 to −1.40 mg/dL; *p* < 0.0001) levels in NAFLD-induced animals with high heterogeneity (TG: *I*^2^ = 84%, *p* < 0.01; TC: *I*^2^ = 74%, *p* < 0.01) (Figure 4A,B). To explore the effects of *Panax ginseng* on hepatic cholesterol metabolism in detail, changes in HDL and LDL levels in NAFLD-induced animals were evaluated. A total of 18 studies and 702 animals measured HDL levels, which were significantly elevated by *Panax ginseng* treatment (SMD: 1.22 mg/dL; 95% CI: 0.59 to 1.85 mg/dL; *p* = 0.0002) with strong heterogeneity (*I*^2^ = 88%, *p* < 0.01) (Figure 4C). Contrary to the results of HDL levels, LDL levels were remarkably decreased by *Panax ginseng* treatment in NAFLD-induced animals (SMD: −1.46 mg/dL; 95% CI: −1.89 to −1.03 mg/dL; *p* < 0.0001) (Figure 4D). These data suggest that *Panax ginseng* may improve hyperlipidemia by regulating hepatic lipid metabolism in NAFLD-induced animals.

### 3.5. Subgroup Analysis

#### 3.5.1. Subgroup Analysis in HFD-Induced NAFLD

**Figure 5:**
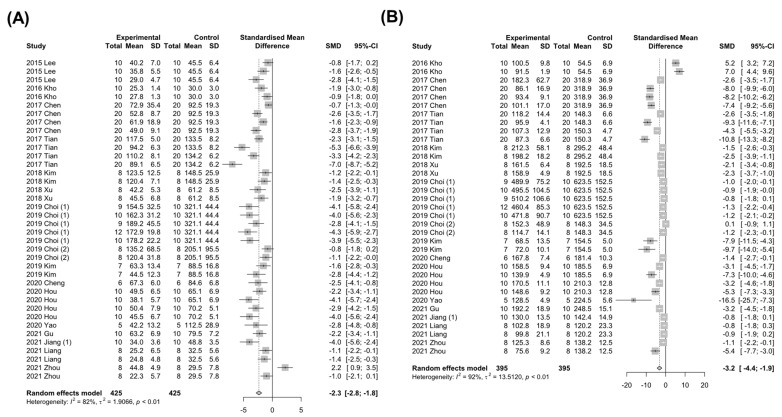
Effects of *Panax ginseng* on liver injury markers in the subgroup analysis of HFD-induced NAFLD. Forest plots for comparison: (**A**) ALT levels [7,26,27,28,30,32,35,36,37,39,40,41,47,48,49,52]. (**B**) AST levels [7,27,28,30,32,35,36,37,39,40,41,47,48,49,52]. SD, standard deviation.

According to the information in the included studies, various animal models were used to induce NAFLD, such as HFD, MCD, the HFHS diet, or *db*/*db* mice. Depending on the method and duration of the experimental model, the severity and mechanisms by which NAFLD is induced may differ. Since chronic HFD feeding is widely used to study NAFLD and is well matched with the pathophysiology of human NAFLD patients [59], subgroup analysis was conducted with studies using the HFD-induced NAFLD model. A total of 16 studies with 850 animals reported systemic ALT levels with high heterogeneity (*I*^2^ = 82%, *p* < 0.01), and the *Panax ginseng* treatment significantly reduced ALT levels in the HFD-induced NAFLD animal model (SMD: −2.33 mg/dL; 95% CI: −2.82 to −1.85 mg/dL; *p* < 0.0001) (Figure 5A). In addition, AST levels were also notably decreased by *Panax ginseng* treatment (SMD: −3.16 mg/dL; 95% CI: −4.43 to −1.90 mg/dL; *p* < 0.0001) (Figure 5B), implying possible involvement of *Panax ginseng* in hepatoprotective effects in HFD-induced NAFLD.

**Figure 6:**
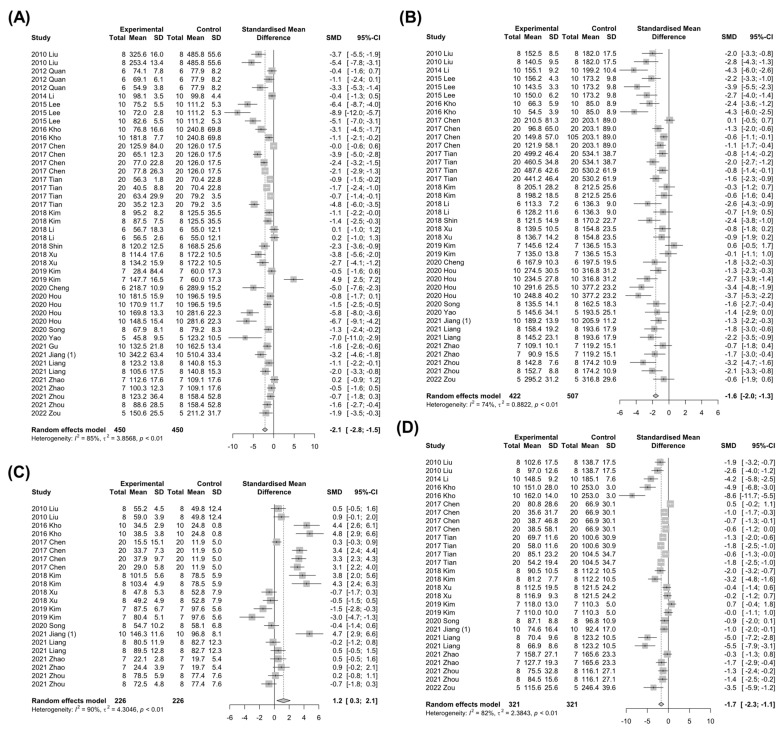
Effects of *Panax ginseng* on markers for hepatic lipid metabolism in the subgroup analysis of HFD-induced NAFLD. Forest plots for comparison: (**A**) TG levels [7,21,22,25,26,27,28,30,32,33,34,35,39,40,41,46,47,48,49,51,52,57]. (**B**) TC levels [7,21,25,26,27,28,30,32,33,34,35,39,40,41,46,47,49,51,52,57]. (**C**) HDL levels [7,21,27,28,32,35,39,46,49,51,52]. (**D**) LDL levels [7,21,25,27,28,30,32,35,39,46,49,51,52,57]. SD, standard deviation.

Changes in serum TG and TC levels of HFD-fed animals were investigated in terms of hepatic lipid metabolism. In line with the results from Figure 4, the subgroup analysis of HFD-induced NAFLD animal models also showed prominently mitigated dyslipidemia by *Panax ginseng* treatment (TG: SMD −2.13 mg/dL, 95% CI −2.75 to −1.51 mg/dL, *p* < 0.0001; TC: SMD −1.61 mg/dL, 95% CI −1.96 to −1.27 mg/dL, *p* < 0.0001) with significant heterogeneity (TG: *I*^2^ = 82%, *p* < 0.01; TC: *I*^2^ = 74%, *p* < 0.01) (Figure 6A,B). In addition, HDL levels were notably elevated (SMD: 1.24 mg/dL; 95% CI: 0.33 to 2.15 mg/dL; *p* = 0.0074) and LDL levels were decreased (SMD: −1.70 mg/dL; 95% CI: −2.32 to −1.08 mg/dL; *p* < 0.0001) by *Panax ginseng* treatment in the subgroup analysis of HFD-induced NAFLD (Figure 6C,D). Based on these results, it might be conceivable that the safeguarding roles of *Panax ginseng* in NAFLD are not limited to a certain experimental model.

#### 3.5.2. Subgroup Analysis According to the Animal Species

**Figure 7:**
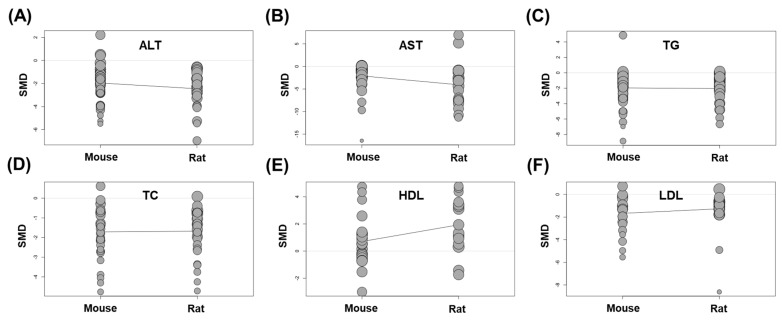
Effects of *Panax ginseng* on NAFLD according to animal species. Bubble plots with the fitted meta-regression lines for comparison: (**A**) ALT levels [7,19,20,23,27,28,29,30,31,32,35,36,37,38,39,40,41,43,44,45,47,48,49,50,52,54,55,56,58]. (**B**) AST levels [19,23,27,28,29,30,31,32,35,36,37,38,39,40,41,44,45,47,48,49,50,52,54,55,56,58]. (**C**) TG levels [7,19,20,21,22,23,24,25,26,27,28,29,30,32,33,34,35,38,39,40,41,44,46,47,48,49,50,51,52,56,58]. (**D**) TC levels [7,19,20,21,23,25,26,27,28,29,30,32,33,34,35,38,39,40,41,44,46,47,49,50,51,52,56,57,58]. (**E**) HDL levels [7,19,21,23,27,28,32,35,38,39,44,46,49,50,51,52,56,58]. (**F**) LDL levels [7,19,21,25,27,28,30,32,35,38,39,46,49,50,51,52,56,57,58]. SD, standard deviation. Each circle indicates each study.

In the present meta-analysis, the animal species used for the study is another variable. Among the total of 41 animal studies, mice or rats were used in 30 (73.2%) or 11 studies (26.8%), respectively. To find out whether the effect of *Panax ginseng* differs depending on the animal species, a meta-regression test was performed by dividing groups of mice and rats. As a result, all factors related to liver injury (ALT and AST) or lipid metabolism (TG, TC, HDL, and LDL) did not show significant linear associations according to animal species (*p* in ALT = 0.184; *p* in AST = 0.069; *p* in TG = 0.867; *p* in TC = 0.909; *p* in HDL = 0.051; *p* in LDL = 0.361) (Figure 7A–F). These data may indicate that the animal species used in the meta-analysis could be a factor in increasing heterogeneity, but did not affect the overall protective effects of *Panax ginseng* in NAFLD-induced animals.

### 3.6. Fasting Blood Glucose

**Figure 8:**
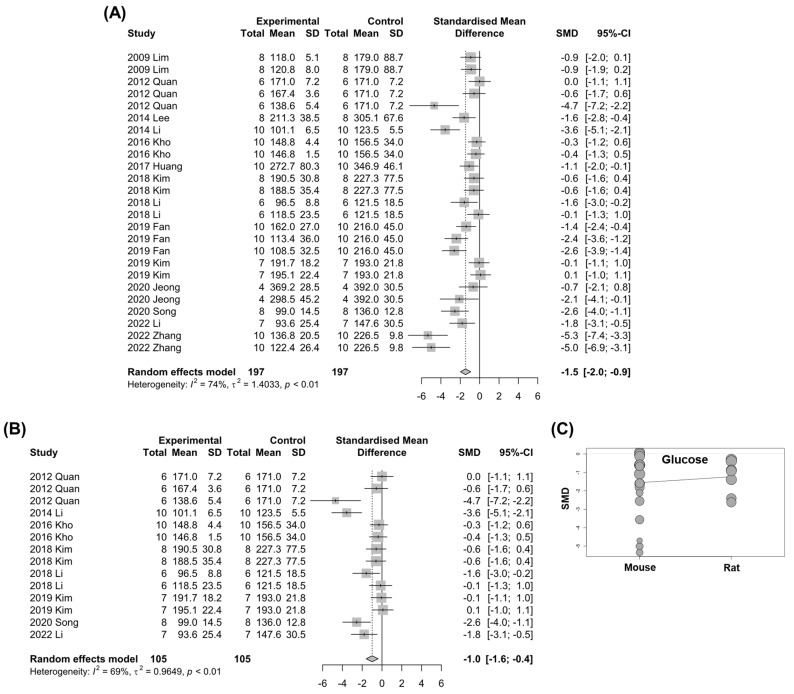
Effects of *Panax ginseng* on fasting blood glucose levels in NAFLD-induced animals. Forest plots for comparison: (**A**) All included studies [19,22,24,25,27,29,32,33,38,39,42,46,53,56]. (**B**) HFD-induced NAFLD subgroup analysis [22,25,27,32,33,39,46,53]. (**C**) A bubble plot with the fitted meta-regression line for the subgroup analysis according to animal species [19,22,24,25,27,29,32,33,38,39,42,46,53,56]. Each circle indicates each study. SD, standard deviation.

Impaired glucose tolerance and insulin resistance are considered crucial hepatic manifestations in the progression of NAFLD [1]. Therefore, studies reporting fasting blood glucose levels were subjected to analysis. A total of 14 studies with 394 animals with high heterogeneity (*I*^2^ = 74%, *p* < 0.01) were analyzed to investigate the overall effect of *Panax ginseng* on fasting blood glucose levels (Figure 8A). As expected, a random-effects model demonstrated that *Panax ginseng* treatment significantly reduced fasting blood glucose levels compared to the control groups (SMD: −1.45 mg/dL; 95% CI: −1.99 to −0.92 mg/dL; *p* < 0.0001) (Figure 8A). In addition, the subgroup analysis with the HFD-induced NAFLD model also presented diminished fasting blood glucose levels by *Panax ginseng* treatment (SMD: −0.99 mg/dL; 95% CI: −1.60 to −0.38 mg/dL; *p* = 0.0014) (Figure 8B). Moreover, there was no difference in the hypoglycemic effect of *Panax ginseng* according to animal species (*p* in fasting blood glucose = 0.599) (Figure 8C).

### 3.7. Publication Bias

Notably, substantial publication bias (*p* < 0.05) was detected in all measured outcomes by the Egger’s bias test of included studies (Supplementary Figure S1). Therefore, although the current study discovered beneficial effects of *Panax ginseng* on NAFLD-induced liver injury, dyslipidemia, and glucose intolerance, these results should be interpreted with caution.

## 4. Discussion

Recent “multiple hit” theories of NAFLD pathogenesis have suggested that both intrahepatic (e.g., inflammation, oxidative stress, mitochondrial dysfunction, and endoplasmic reticulum (ER) stress) and extrahepatic (e.g., gut microbiome or adipokines) mechanisms cooperatively contribute to NAFLD development [3]. Because of the heterogeneous nature of NAFLD pathogenesis, there is still no approved drug for NAFLD, even though numerous clinical trials are ongoing. *Panax ginseng* is a traditional herbal medicine in eastern countries that exerts anti-oxidative, anti-inflammatory, and anti-microbial effects. Due to these beneficial functions of *Panax ginseng*, the preclinical study with *Panax ginseng* has been performed on various diseases, such as liver diseases, metabolic disorders, cardiovascular diseases, and neurological diseases, in which oxidative stress and inflammatory responses play important roles [9,10]. However, although many publications were investigating the role of *Panax ginseng* in NAFLD, to the best of our knowledge, there is no systematic review and meta-analysis related to this topic. Due to this limited information on the efficacy of *Panax ginseng* for NAFLD, only a small-scale clinical study has been conducted with *Panax ginseng* in patients with NAFLD [60].

In the systematic review and meta-analysis of a preclinical study, it is important to carefully set the primary outcomes so that the results can be applied to the clinic. Although a reliable endpoint that can effectively predict the outcome of patients is still lacking, most of the ongoing NAFLD clinical trials set the markers for liver injury (ALT and AST), lipid metabolism (TG, TC, HDL, and LDL), and hyperglycemia (fasting blood glucose) as primary outcomes. In line with this clinical setting, the present study concisely analyzed the efficacy of *Panax ginseng* on NAFLD-related liver injury, dyslipidemia, and glucose tolerance in the preclinical studies using the above parameters as primary outcomes.

As a result, *Panax ginseng* treatment significantly diminished NAFLD-induced systemic ALT, AST, TG, TC, LDL, and fasting blood glucose levels while elevating HDL levels in animals. These results are partially consistent with a previous clinical trial in which blood ALT, gamma-glutamyl transferase, and TG levels of patients with NAFLD were significantly reduced by 30 days of 2 g day^−1^ treatment with Korean red ginseng compared to placebo controls [60]. In this study, TC levels were significantly reduced compared to the baseline in NAFLD patients treated with Korean red ginseng, but there was no difference when compared to the placebo controls. In addition, AST and fasting glucose levels were not changed by the Korean red ginseng treatment. However, since the participants in the above clinical trial were also administered milk thistle extracts (450 mg d^−1^; Legalon^®^), further clinical trials are needed to demonstrate the protective effects of *Panax ginseng* in patients with NAFLD. Interestingly, the current meta-analysis acquired promising results from highly heterogeneous studies on the dose (0.4 mg kg^−1^ day^−1^ to 1000 mg kg^−1^ day^−1^), duration (3 days to 16 weeks), and route of *Panax ginseng* treatment. It may imply that *Panax ginseng* has multiple downstream targets in NAFLD. Although the underlying mechanisms could not be delineated by this study, included articles have suggested several protective mechanisms mediated by *Panax ginseng* treatment against NAFLD. First, in hepatocytes, *Panax ginseng* treatment suppressed NAFLD-induced cellular stress responses, such as oxidative stress [23,50], ER stress [35,47], and mitochondrial dysfunction [26]. In addition, the administration of *Panax ginseng* has beneficial effects on hepatic energy metabolism by activating AMP-activated protein kinase [22,29,56,58] but inhibiting the mammalian target of rapamycin complex 1 [37], resulting in the normalization of hepatic glucose [30] and lipid metabolism [32,33,34,41] in NAFLD. In the hepatic microenvironment, *Panax ginseng* reduced inflammatory responses by preventing activation of the NLR family pyrin domain containing 3 (NLRP3) inflammasome and nuclear factor kappa-light-chain-enhancer of activated B cells (NF-κB), thereby suppressing gene and protein expression levels of interleukin (IL)-1β, IL-6, and tumor necrosis factor-α [35,36,38,49,55]. In the extrahepatic milieu, *Panax ginseng* rebounded adiponectin production by adipocytes [53] and restricted the expansion of adipose tissue by inhibiting angiogenesis [24]. In addition, recent studies have suggested that the beneficial roles of *Panax ginseng* on hepatic lipid metabolism may be derived from the modulation of the gut microbiome [51,57,58].

As such, the various protective mechanisms of *Panax ginseng* may suggest that it could help treat NAFLD patients with high heterogeneity in their pathophysiology. Indeed, in the subgroup analyses of the current study, the therapeutic effects of *Panax ginseng* in NAFLD-induced animals were not dependent on the experimental models or animal species used. However, although the beneficial effects of *Panax ginseng* treatment were found in NAFLD-induced animals, there was a publication bias in all outcomes in Egger’s test. Publication bias is the phenomenon in which the results of experiments determine the likelihood of publication, often overinterpreting positive results. Despite the significant publication bias of the present study, multiple lines of evidence support the protective effect of *Panax ginseng* in NAFLD. Furthermore, in the case of animal experiments to investigate the efficacy of *Panax ginseng*, publication might be difficult if the effect of treatment was unsatisfactory. Therefore, although the present study demonstrated the protective effects of *Panax ginseng* on NAFLD-induced animals, all results of the primary outcomes should be interpreted with caution, and more well-designed preclinical and clinical studies are required.

Despite the present meta-analysis providing rigorous information on the efficacy of *Panax ginseng* on NAFLD, there are several limitations. First of all, the most critical limitation of this study is that the efficacy of *Panax ginseng* was evaluated only in preclinical animal models. Although the various NAFLD models used in the included literature can mimic the pathobiology of human NAFLD patients in part, there are significant discrepancies in the pathogenesis and effects of *Panax ginseng* in NAFLD between animals and patients. In addition, due to the nature of animal experiments, included studies had greater heterogeneity with various experimental conditions compared to patients. Another limitation is that the current study could not clarify the efficient components, doses, durations, and routes of *Panax ginseng* in NAFLD in detail. As this study detected constant favorable outcomes, including liver injury, from all included studies, *Panax ginseng* may have therapeutic potential in NAFLD. However, a future study that directly compares the components-, dose-, duration-, and route-dependent efficacy of *Panax ginseng* in NAFLD will be necessary for its therapeutic application.

## 5. Conclusions

The present study demonstrated that the treatment of *Panax ginseng* significantly ameliorated liver injury and disturbances of lipid and glucose metabolism in NAFLD-induced animals, regardless of the experimental model. Although the protective mechanisms of *Panax ginseng* on NAFLD remain unknown, this meta-analysis suggested that *Panax ginseng* has both intra- and extrahepatic beneficial effects. Because there was considerable heterogeneity and publication bias in the included studies, the results should be interpreted with caution.

## Figures and Tables

**Table 1:** Literature searching strategy.

PubMed
“ginsengs” (All Fields) OR “panax” (MeSH Terms) OR “panax” (All Fields) OR “ginseng” (All Fields) OR “ginsengs” (All Fields)) AND (“fatty liver” (MeSH Terms) OR (“fatty” (All Fields) AND “liver” (All Fields)) OR “fatty liver” (All Fields)
Web of Science
(ginseng) OR (panax) (fatty liver) OR (non-alcoholic fatty liver disease) #1 AND #2
Cochrane Library
(ginseng) OR (panax) (fatty liver) OR (non-alcoholic fatty liver disease) #1 AND #2

“#” means number.

**Table 2:** Overall characteristics of included literature.

Study	Country	Animal (Sex)	NAFLD Model	Component	Dose	Route and Times
2009 Lim [19]	Republic of Korea	OLETF rat (male)	32 weeks	Ginsam (a vinegar extract from *Panax ginseng*)	300 mg kg^−1^	p.o., daily (last 8 weeks)
2010 Kwak [20]	Republic of Korea	Sprague-Dawley rat (male)	Triton WR1339 (i.v., 150 mg kg^−1^)	Korean red ginseng	100, 500, or 1000 mg kg^−1^	p.o., daily (for 3 days before Triton)
2010 Liu [21]	China	Kunming mouse (female)	HFD (8 weeks)	Protopanaxdiol	0.02% or 0.05% in the diet	p.o., daily
2012 Quan [22]	Republic of Korea	C57BL/6J mouse (undefined)	HFD (6 weeks)	Ginsenoside Re	5, 10, or 20 mg kg^−1^	p.o., daily (last 3 weeks)
2013 Hong [23]	Republic of Korea	OLETF rat (male)	12 months	Korean red ginseng	200 mg kg^−1^ in the diet	p.o., daily (last 8 weeks)
2014 Lee [24]	Republic of Korea	*db*/*db* mouse (female)	13 weeks	Total ginsenosides	5% of the diet	p.o., daily
2014 Li [25]	United States	C57BL/6J mouse (male)	HFD (15 weeks)	Chinese ginseng	500 mg kg^−1^	p.o., daily
2015 Lee [26]	Republic of Korea	C57BL/6 mouse (male)	HFD (10 weeks)	Ginseng cambial meristematic cells	75, 150, or 300 mg kg^−1^	p.o., daily
2016 Kho [27]	Republic of Korea	Sprague-Dawley rat (male)	High-fructose diet (8 weeks)	Red ginseng and *Polygoni Multiflori* Radix (1:1 ratio)	100 or 300 mg kg^−1^	p.o., daily (last 6 weeks)
2017 Chen [28]	China	Sprague-Dawley rat (male)	HFD (11 weeks)	Chinese ginseng saponins	50 mg kg^−1^	i.p., daily (last 10 weeks)
				Ginsenoside Compound K	3 mg kg^−1^	
				Ginsenoside Rh1	3 mg kg^−1^	
				Compound K + Rh1	3 mg kg^−1^	
2017 Huang [29]	China	*db/db* mouse (male)	10 weeks	Ginsenoside Rb2	10 mg kg^−1^	i.p., daily (last 4 weeks)
2017 Tian [30]	China	Sprague-Dawley rat (male)	HFD (12 weeks)	Ginsenoside Rg1	25 or 50 mg kg^−1^	p.o., daily (last 4 weeks)
			HFD (16 weeks)		25 or 50 mg kg^−1^	p.o., daily (last 8 weeks)
2018 Jeong [31]	Republic of Korea	C57BL/6 mouse (male)	MCD diet (13 weeks)	Korean red ginseng	100, 200, or 400 mg kg^−1^	p.o., daily
2018 Kim [32]	Republic of Korea	C57BL/6J mouse (undefined)	HFD (12 weeks)	Ginseng seed oil	250 or 500 mg kg^−1^	p.o., daily
2018 Li [33]	Republic of Korea	C57BL/6 mouse (male)	HFD (16 weeks)	Fermented ginseng root	Not indicated, supplemented with a diet	p.o., daily
				Fermented ginseng berry		
2018 Shin [34]	Republic of Korea	C57BL/6 mouse (male)	HFD (8 weeks)	Ginseng extract	5% in the diet	p.o., daily
2018 Xu [35]	China	C57BL/6 mouse (female)	HFD (20 weeks)	Ginsenoside Rg1	20 or 40 mg kg^−1^	p.o., daily (last 4 weeks)
2019 Choi (1) [36]	Republic of Korea	C57BL/6 mouse (male)	Western diet (12 weeks)	GBCK25	10, 20, 100, 200, or 400 mg kg^−1^	p.o., daily
2019 Choi (2) [37]	Republic of Korea	C57BL/6N mouse (male)	Fast-food diet (14 weeks)	Fermented Korean red ginseng	100 or 300 mg kg^−1^	p.o., daily (last 8 weeks)
2019 Fan [38]	China	Sprague Dawley rats (undefined)	HFHS diet (16 weeks)	Ginsenoside Rg1	10, 25, or 50 mg kg^−1^	p.o., daily (last 4 weeks)
2019 Kim [39]	Republic of Korea	C57BL/6 mouse (male)	HFD (6 weeks)	Korean red ginseng	0.4 mg kg^−1^	p.o., daily
				Korean red ginseng and probiotics	0.4 mg kg^−1^ + 0.03 mg kg^−1^	
2020 Cheng [40]	China	C57BL/6J mouse (undefined)	HFD (16 weeks)	Ginsenoside Rg2	10 mg kg^−1^	i.p., daily (last 4 weeks)
2020 Hou [41]	China	Sprague Dawley rats (undefined)	HFHS diet (14 weeks)	Ginsenoside Rg1	30 or 60 mg kg^−1^	p.o., daily (last 4 weeks)
						p.o., daily (last 8 weeks)
2020 Jeong [42]	Republic of Korea	*db/db* mouse (male)	10 weeks	Black ginseng	100 or 900 mg kg^−1^	p.o., daily (last 5 weeks)
2020 Lee [43]	Republic of Korea	C57BL/6J mouse (male)	MCD diet (6 weeks)	Ginsenoside Rg3	15 or 30 mg kg^−1^	p.o., daily (last 3 weeks)
2020 Park [44]	Republic of Korea	ICR mouse (male)	High-fat high-fructose diet (17 weeks)	Black ginseng	0.5%, 1%, or 2% in the diet	p.o., daily (last 8 weeks)
2020 Qi [45]	China	C57BL/6J mouse (male)	Intraperitoneal D-galactose (120 mg kg^−1^ day^−1^) injection (6 weeks)	Ginsenoside Rg1	40 mg kg^−1^	i.p., daily (last 4 weeks)
2020 Song [46]	China	C57BL/6J mouse (male)	HFD (24 weeks)	Ginsenoside Rb1	10 mg kg^−1^	p.o., daily (last 8 weeks)
2020 Yao [47]	China	C57BL/6J mouse (male)	HFD (12 weeks)	*Panax ginseng* root extract	120 mg kg^−1^	p.o., daily (last 4 weeks)
2021 Gu [48]	China	Sprague Dawley rats (male)	HFHS diet (26 weeks)	Ginsenoside Rg1	100 mg kg^−1^	p.o., daily (last 8 weeks)
2021 Jiang (1) [49]	Pakistan	C57BL/6 mouse (male)	HFD (6 weeks)	Black ginseng extract	100 mg kg^−1^	p.o., daily
2021 Jiang (2) [50]	China	*db/db* mouse (male)	8 weeks	Ginsenoside Re	30 mg kg^−1^	p.o., daily
2021 Liang [7]	China	C57BL/6J mouse (male)	HFD (12 weeks)	*Panax ginseng*	100 or 200 mg kg^−1^	p.o., daily
2021 Zhao [51]	China	Sprague Dawley rats (male)	HFD (8 weeks)	Fermented *Panax ginseng*	200 or 400 mg kg^−1^	p.o., daily
2021 Zhou [52]	China	C57BL/6J mouse (male)	HFD (8 weeks)	Ginsenoside F2	50 or 100 mg kg^−1^	p.o., daily (last 4 weeks)
2022 Li [53]	China	C57BL/6J mouse (male)	HFD (14 weeks)	Ginsenoside Rb1	10 mg kg^−1^	i.p., daily (last 2 weeks)
2022 Wei (1) [54]	China	C57BL/6J mouse (male)	MCD diet (6 weeks)	Black ginseng extracts	300, 600, or 900 mg kg^−1^	p.o., daily (last 3 weeks)
2022 Wei (2) [55]	China	C57BL/6 mouse (male)	Western diet and sugar water and carbon tetrachloride (2 mL kg^−1^, once a week) (12 weeks)	Black ginseng extracts	300, 600, or 900 mg kg^−1^	p.o., daily (last 4 weeks)
2022 Zhang [56]	China	C57BL/6 mouse (male)	30% fructose in water (4 weeks)	Ginsenoside Compound K	30 or 60 mg kg^−1^	p.o., daily
2022 Zou [57]	China	C57BL/6J mouse (male)	HFD (12 weeks)	Ginsenoside Rb1	120 mg kg^−1^	p.o., daily (last 4 weeks)
2023 Ren [58]	China	Sprague Dawley rats (male)	High-glucose high-fat diet (8 weeks) and streptozotocin (35 mg kg^−1^)	Rg1-enriched polysaccharide	100 or 300 mg kg^−1^	p.o., daily

HFD, high-fat diet; HFHS, high-fat high-sucrose; ICR, institute of cancer research; i.p., intraperitoneal injection; MCD, methionine-choline deficient diet; NAFLD, nonalcoholic fatty liver; OLETF, Otsuka Long-Evans Tokushima; p.o., per os.

## Data Availability

Published systematic review and PROSPERO (CRD42023381574).

## Supplementary Materials

The following supporting information can be downloaded at: https://www.mdpi.com/article/10.3390/nu15030721/s1, Figure S1: Funnel plots of the measured outcomes reflect publication bias.
