# Supplementary material for: The Efficacy of Panax ginseng for the Treatment of Nonalcoholic Fatty Liver Disease: A Systematic Review and Meta-Analysis of Preclinical Studies
Source: Nutrients. 2023 Jan 31;15(3):721. doi: 10.3390/nu15030721 (PMC9919883; doi:10.3390/nu15030721)
Supplement: Supplementary file 1 [file nutrients-15-00721-s001.zip › nutrients-2175054-supplementary.pdf]

nutrients

The Efficacy of *Panax ginseng* for the Treatment of Nonalcoholic Fatty Liver Disease: A Systematic Review and Meta-Analysis of Preclinical Studies

Keungmo Yang, Hee-Hoon Kim, Young-Ri Shim and Myeong Jun Song

Supplementary Materials

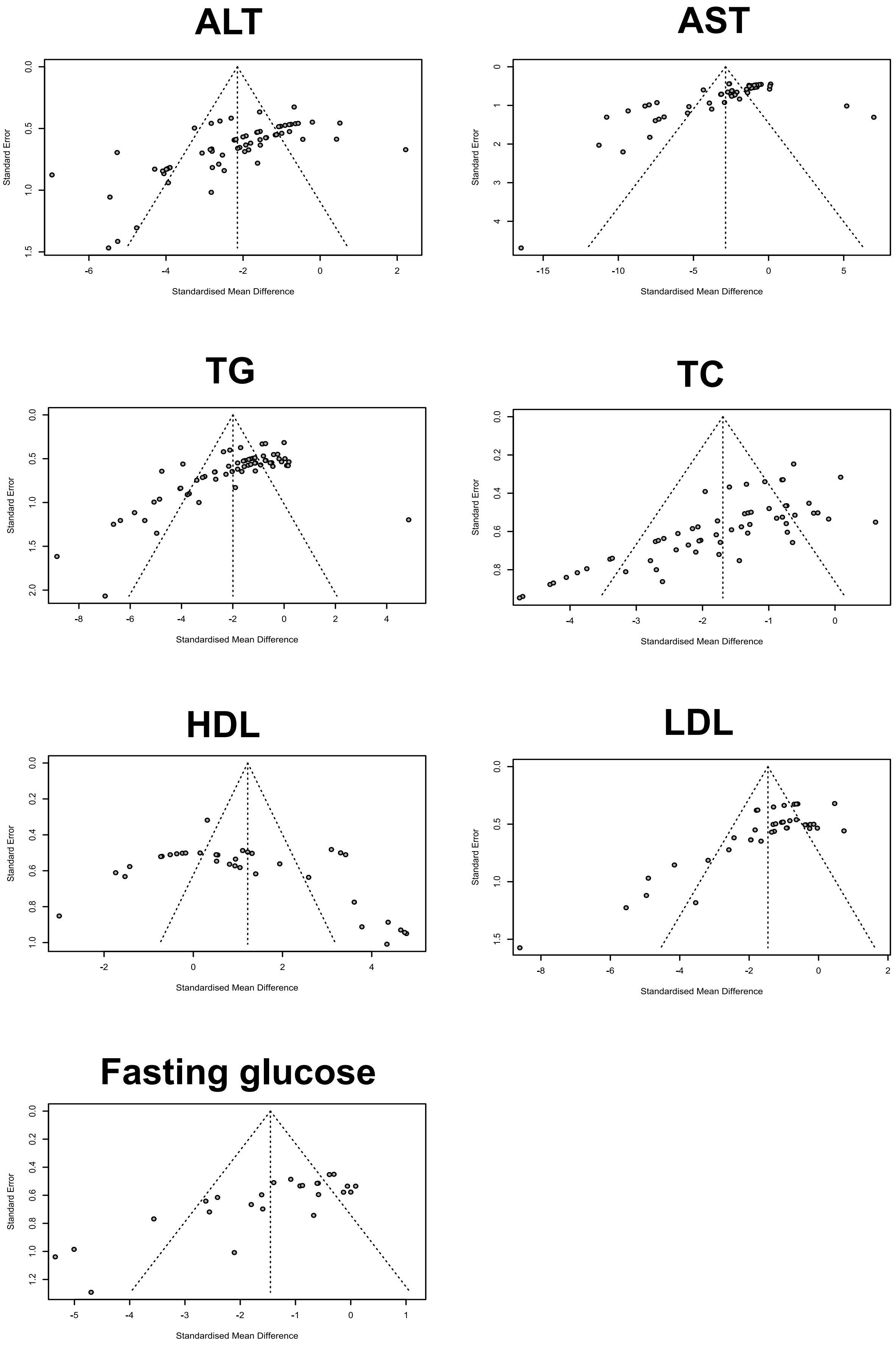

Supplementary Figure S1. Funnel plots of the measured outcomes reflect publication bias.
